# Considering the Spatial Layout Information of Bag of Features (BoF) Framework for Image Classification

**DOI:** 10.1371/journal.pone.0131164

**Published:** 2015-06-29

**Authors:** Guangyu Mu, Ying Liu, Limin Wang

**Affiliations:** Dept. of Management Science and Information Engineering, Jilin University of Finance and Economics, Changchun, China; University of Akron, UNITED STATES

## Abstract

The spatial pooling method such as spatial pyramid matching (SPM) is very crucial in the bag of features model used in image classification. SPM partitions the image into a set of regular grids and assumes that the spatial layout of all visual words obey the uniform distribution over these regular grids. However, in practice, we consider that different visual words should obey different spatial layout distributions. To improve SPM, we develop a novel spatial pooling method, namely spatial distribution pooling (SDP). The proposed SDP method uses an extension model of Gauss mixture model to estimate the spatial layout distributions of the visual vocabulary. For each visual word type, SDP can generate a set of flexible grids rather than the regular grids from the traditional SPM. Furthermore, we can compute the grid weights for visual word tokens according to their spatial coordinates. The experimental results demonstrate that SDP outperforms the traditional spatial pooling methods, and is competitive with the state-of-the-art classification accuracy on several challenging image datasets.

## Introduction

Image classification plays a significant role in the computer vision research. The recent state-of-the-art image classification pipeline consists of two major parts: 1) the image representation, e.g., bag of features (BoF) [1–3] and spatial pyramid matching (SPM) [4]; 2) the classifier, e.g., support vector machines (SVMs) and its variants [5, 6]. Nowadays, developing discriminative image representation is challenging for image classification.

Referring to the bag of words (BoW) used in textual information retrieval, the BoF method has been widely used for image representation [1–3]. The standard BoF model first extracts the local feature, e.g., the SIFT descriptor, from all images, and then uses cluster algorithms or vector quantization methods to transform local features into a visual vocabulary, where each cluster delegates a visual word type. Thus, BoF can describe the images as orderless collections of the visual word. The representative extensions of BoF include the geometric correspondence search [7, 8], the discriminative vocabulary learning [9–12], and the constrained coding methods [5, 13].

**Fig 1:**
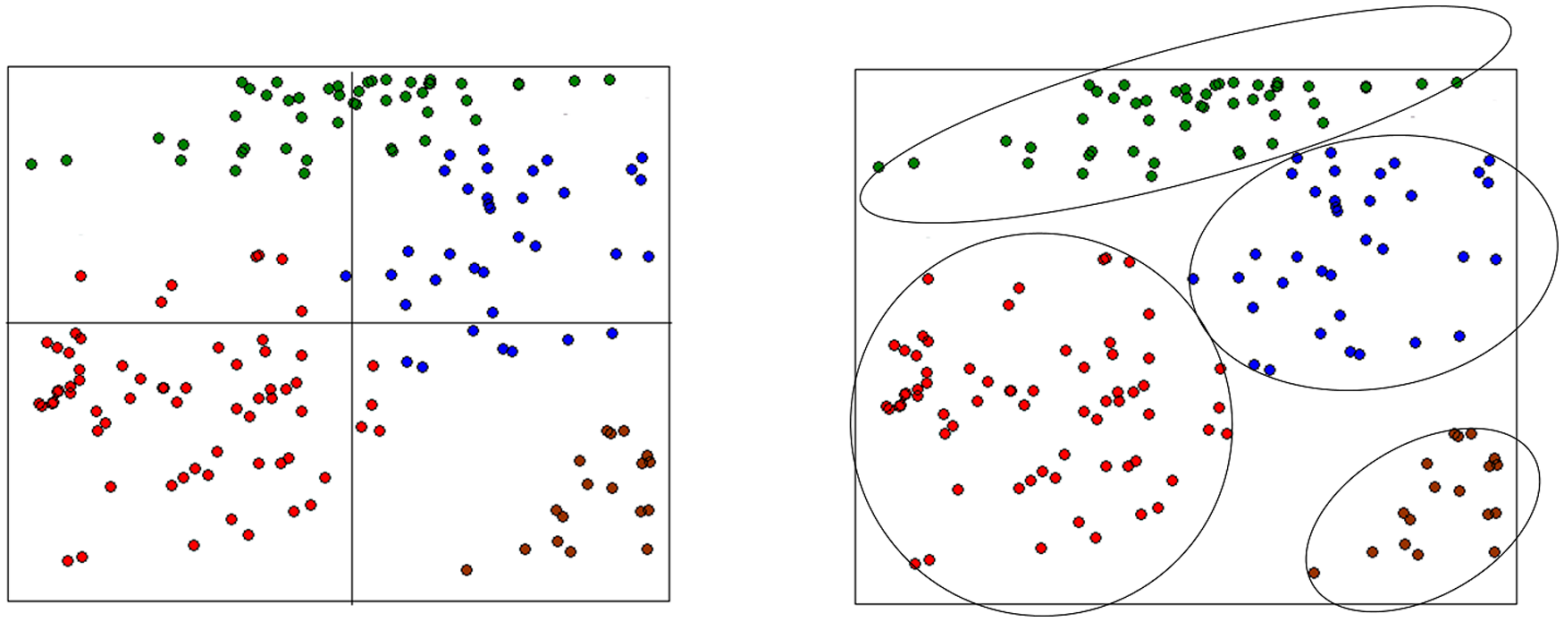
An example of the spatial layout of a certain visual word type. SPM (left) partitions the image into 4 regular grids, and SDP (right) partitions the image into 4 flexible grids. We argue that the grids from SDP are more reasonable.

**Fig 2:**
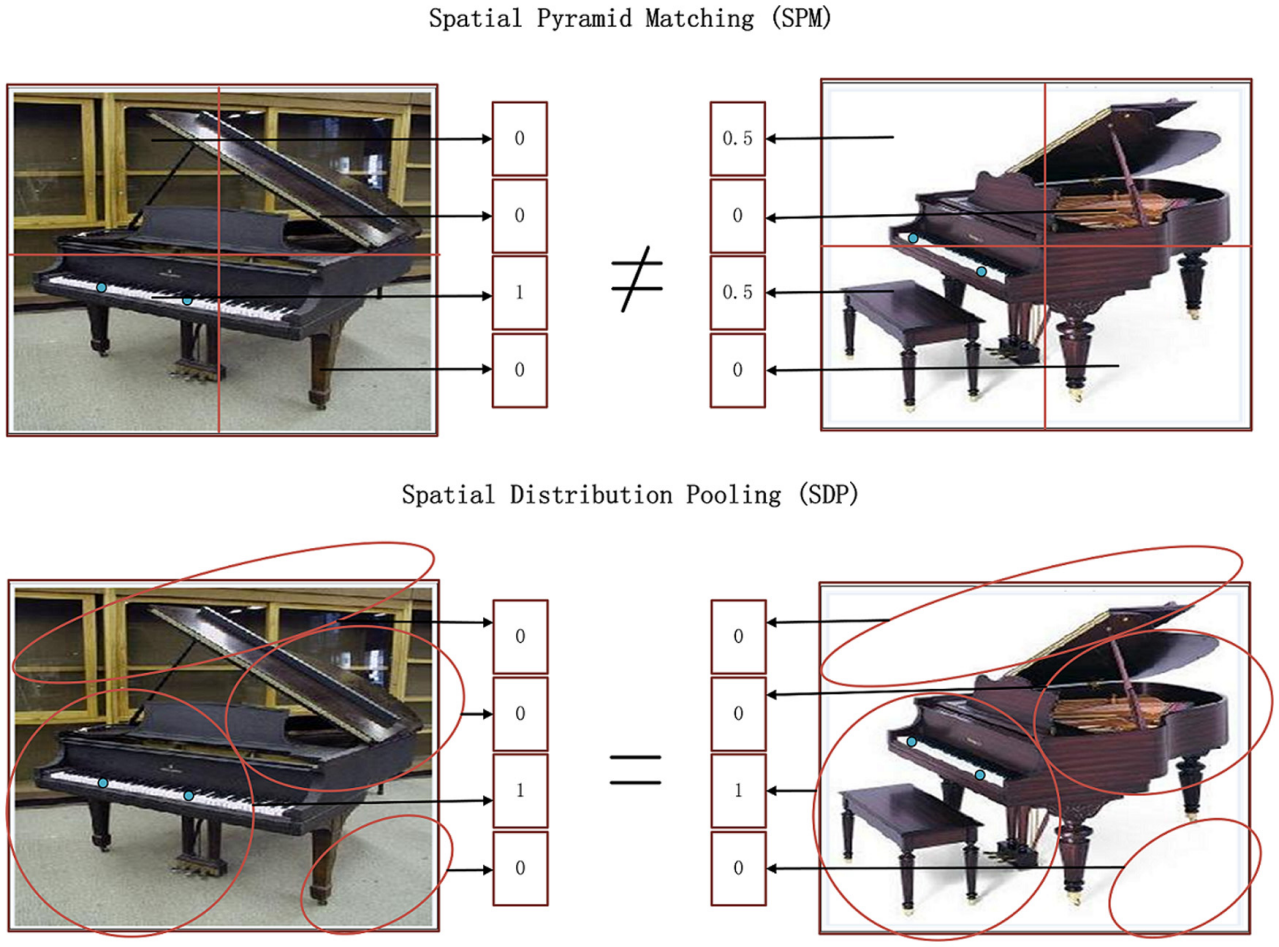
The image-level representation constructed by SPM and SDP following Fig 1. The blue circle is the visual word described in Fig 1. For two similar piano images, SDP generates two equal image-level feature vectors, but SPM generates inconsistent image-level feature vectors.

To further improve BoF by considering the spatial layout information, the authors of [4] propose a downstream SPM method for BoF. After generating the visual vocabulary, SPM partitions the image into a set of regular grids at different levels and concatenates histograms of visual words from each grid. Empirical results show that SPM can significantly improve the classification performance, however, it assumes that the spatial layout of all visual words obey the uniform distribution over these regular grids. This generates a conflict to the intuition that different visual words should obey different spatial layout distributions. To address this problem, we suggest a novel spatial distribution pooling (SDP) algorithm to improve SPM. In SDP, we develop an extension model of Gauss mixture model (GMM), and use this model to estimate the spatial layout distribution for each visual word type. SDP can generate a set of flexible grids rather than regular grids from the traditional SPM. As the example shown in Fig 1, SDP can generate more reasonable grids than SPM, resulting in (i.e., Fig 2) more consistent image-level representation than SPM. Furthermore, SDP can compute the grid weights for visual word tokens according to their spatial coordinates. A number of experiments have been conducted to evaluate SDP. The experimental results demonstrate that SDP outperforms the existing spatial pooling methods.

The reminder of this paper is organized as follow: In Section 2, we introduce the framework of the proposed algorithm. In Section 3, we report and discuss the experimental results. In Section 4, conclusions are discussed.

## Proposed Algorithm

In this section, we first review the popular SPM-based image classification system, and then introduce the proposed SDP algorithm.

Given a visual vocabulary with *V* visual words, let $\vec{N}=\left[n_{1},n_{2},\cdots ,n_{V}\right]$, where *n*_*v*_ is the number of times that visual word *v* has occurred in the training images and $N=\sum_{v=1}^{V}n_{v}$. Let $C_{v}=\left\{\vec{c_{1}^{v}},\vec{c_{2}^{v}},\cdots ,\vec{c_{n_{v}}^{v}}\right\}$ be the full spatial layout (i.e., spatial coordinate) set for visual word *v* (as shown in Fig 1), where $\vec{c_{i}^{v}}=\left[x_{i}^{v},y_{i}^{v}\right]$.

### SPM-based Image Classification System

**Fig 3:**
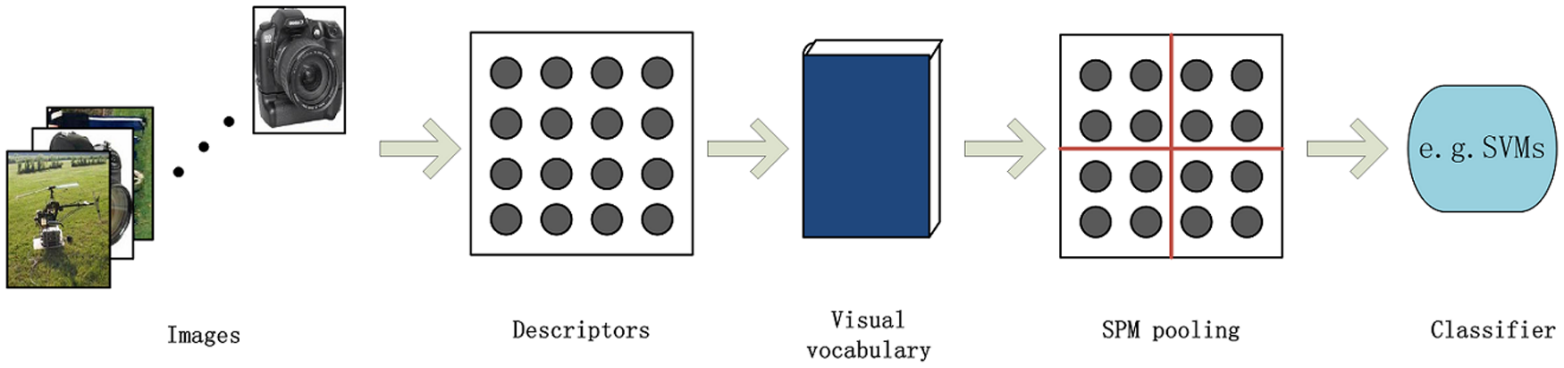
The flowchart of the SPM-based image classification system.

We introduce the traditional flowchart of the SPM-based image classification system. As shown in Fig 3, this system extracts local features, e.g., SIFT and DHOG [1, 14] descriptors, from all images, and then codes these local features into a visual vocabulary using clustering algorithms or vector quantization methods [5, 9–13]. For each image, it transforms the local features into visual words according to the visual vocabulary, and then generates its image-level feature vector using SPM. After sweeping all images, the traditional algorithm, e.g., SVMs, is commonly used to train the classifier.

**Fig 4:**
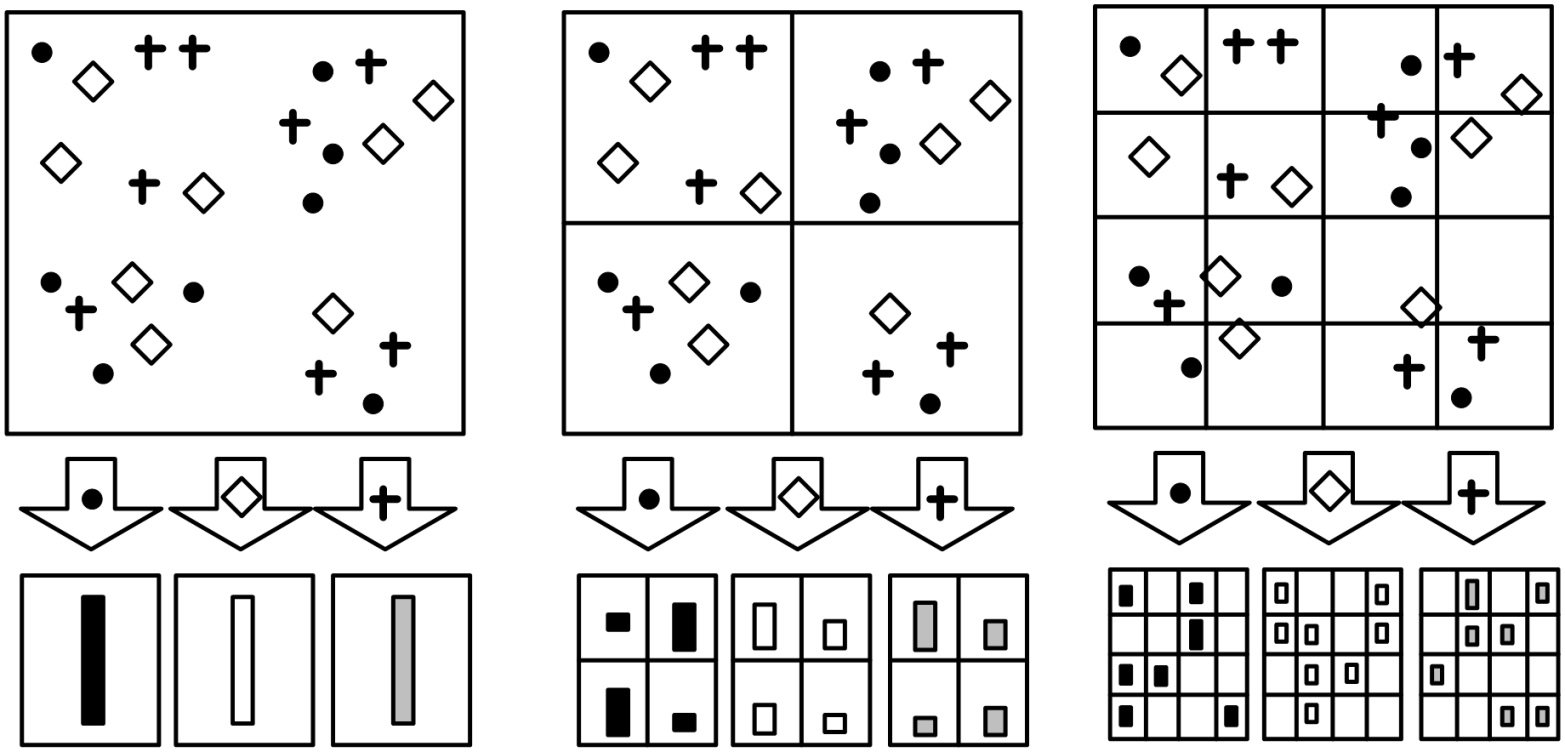
An example for SPM.

In this image classification system, SPM is used to capture the spatial layout information. For clarity, we illustrate an example of SPM with 2^*l*^ × 2^*l*^ grids each level, where the level *l* is set to be 0, 1, 2. As shown in Fig 4, suppose that the visual vocabulary contains three visual word types (i.e., *V* = 3), which are indicated by circles, diamonds and crosses. Following the above setting, SPM divides the image at three levels, and then count the visual word histograms for each grid in each level. Concatenating all visual word histograms together, we can finally construct an image-level feature vector. Considering this example, each image corresponds to a 63-dimensional (i.e., $V\times \sum_{l=0}^{2}2^{l}=63$) feature vector.

### Spatial Distribution Pooling

SPM rigidly partitions the image into several regular grids, and assumes that the spatial layout of all visual words obey the uniform distribution over these grids. That is to say, each visual word in SDP occurs in the regular grids in each level following equal probability. However, this generates a conflict to the intuition that different visual words should obey different spatial layout distributions. To address this problem, we propose a spatial distribution pooling (SDP) algorithm. Inspired by the idea of generative Bayesian model [15, 16], we develop an extension model of GMM (e-GMM) to describe spatial layout distributions of visual words. We assume that visual words are independently drawn. For each visual word *v*, its spatial layout $\vec{\phi_{v}}$ is a multinomial distribution over *K* latent grids, drawn from the Dirichlet prior *β*. Each latent grid *k* is a bivariate Gaussian distribution with respect to the spatial coordinate of the visual word token, where $\mu_{k}^{v}$ is the expectation and $\Sigma_{k}^{v}$ is the covariance matrix. Formally, the spatial coordinate generative process for visual word *v* is as follows:

1. Choose $\vec{\phi_{v}}∼Dirichlet(\beta )$
2. For each of the *n*_*v*_ visual word *v*:
  - Choose a latent grid: $z_{n}∼Multinomial(\vec{\phi_{v}})$
  - Sample the spatial coordinate $\vec{c_{n}^{v}}$ of the *n*-th visual word *v* from $Gauss(\mu_{z_{n}}^{v},\Sigma_{z_{n}}^{v})$.

where

$$\begin{array}{c} Gauss\left(\vec{c^{v}},\mu_{k}^{v},\Sigma_{k}^{v}\right)=\frac{\exp[-\frac{1}{2}{\left(\vec{c^{v}}-\mu_{k}^{v}\right)}^{T}{\left(\Sigma_{k}^{v}\right)}^{-1}(\vec{c^{v}}-\mu_{k}^{v})]}{(2\pi ){\left|\Sigma_{k}^{v}\right|}^{-1}} \end{array}$$ (1)

**Fig 5:**
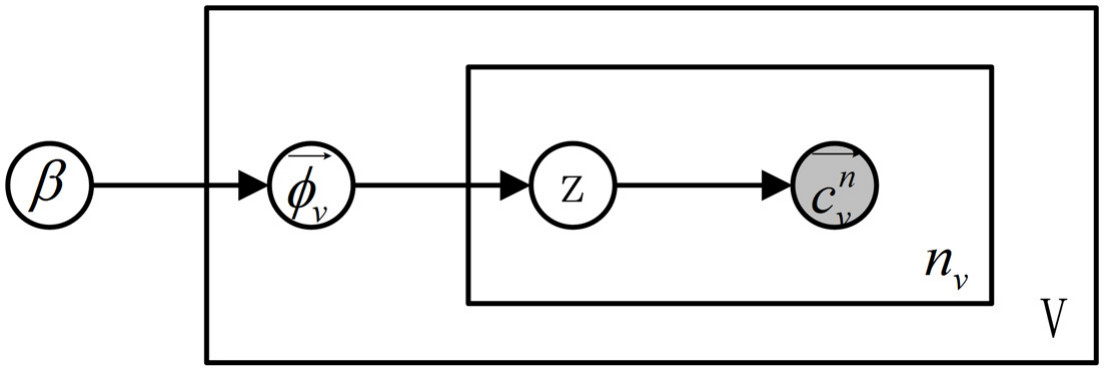
The graphical model representation of e-GMM.

The graphical model representation of e-GMM is given in Fig 5. SDP is more flexible compared to SPM. Under e-GMM, SDP can assign each visual word to a latent grid according to its spatial coordinate, instead of a regular grid. For each image, we can construct its image-level feature vector by concatenating all visual word histograms of latent grids together.

### Inference and Estimation

In this section, we discuss the two inherent issues of e-GMM: 1) Inference: if the parameters of e-GMM (i.e., *β*, ${\left\{\vec{\phi_{v}}\right\}}_{v=1}^{V}$, ${\left\{\mu_{k}^{v}\right\}}_{k=1,v=1}^{k=K,v=V}$ and ${\left\{\Sigma_{k}^{v}\right\}}_{k=1,v=1}^{k=K,v=V}$) are known, given a visual word *v* with spatial coordinate $\vec{c^{v}}$, we want to infer its corresponding latent grid; 2) Estimation: given a number of visual word *v* with the spatial coordinate set *C*_*v*_, we want to estimate model parameters with respect to visual word *v* (i.e., $\vec{\phi_{v}}$, ${\left\{\mu_{k}^{v}\right\}}_{k=1}^{K}$ and ${\left\{\Sigma_{k}^{v}\right\}}_{k=1}^{K}$).

#### Inference

The inferential problem is to compute the posterior distribution of the grid assignment given a visual word *v* with spatial coordinate $\vec{c^{v}}$. It can be computed as follows:

$$\begin{array}{c} p\;(z=k|\vec{c_{v}},\vec{\phi_{v}},\mu^{v},\Sigma^{v})\propto \phi_{v,k}Gauss\;(\vec{c_{v}},\mu_{k}^{v},\Sigma_{k}^{v}) \end{array}$$ (2)

We consider the posterior as gird weights of this visual word token. We sort these *K* grid weights, and use the top *M* (where *M* ∈ {1, 2, ⋯, *K*}) values to accumulate histograms of visual word *v* in the corresponding latent grids. The final grid weight values used are computed by:

$$\begin{array}{c} p\;(z=k_{m}|\vec{c_{v}},\vec{\phi_{v}},\mu^{v},\Sigma^{v})=\frac{p\;(z=k_{m}|\vec{c_{v}},\vec{\phi_{v}},\mu^{v},\Sigma^{v})}{\sum_{i=1}^{M}p\;(z=k_{i}|\vec{c_{v}},\vec{\phi_{v}},\mu^{v},\Sigma^{v})} \end{array}$$ (3)

where *k*_*m*_, as well as *k*_*i*_, is one of the top *M* latent grids. For clarity, we illustrate an example of *M* = 3 setting. Suppose that there is a visual word token *v* assigned three grids {1, 2, 3} with grid weights {0.1, 0.3, 0.6}. We consider that the visual word *v* occurs in the latent grid 1 0.1 times, the latent grid 2 0.3 times and the latent grid 3 0.6 times.

#### Estimation

For each visual word *v*, given $C_{v}=\left\{\vec{c_{1}^{v}},\vec{c_{2}^{v}},\cdots ,\vec{c_{n_{v}}^{v}}\right\}$ we wish to estimate the e-GMM parameters $\vec{\phi_{v}}$, ${\left\{\mu_{k}^{v}\right\}}_{k=1}^{K}$ and ${\left\{\Sigma_{k}^{v}\right\}}_{k=1}^{K}$. This can be achieved by maximizing the likelihood:

$$\begin{array}{c} p\;(C_{v}|\beta ,\mu^{v},\Sigma^{v})=\int p\;(\vec{\phi_{v}}|\beta )\prod_{n=1}^{n_{v}}\sum_{z}p\;(z_{n}|\vec{\phi_{v}})\;p\;(\vec{c_{n}^{v}}|z_{n},\mu^{v},\Sigma^{v})\;d\vec{\phi_{v}} \end{array}$$ (4)

where *z* is the grid assignments; $\mu^{v}={\left\{\mu_{k}^{v}\right\}}_{k=1}^{K}$ and $\Sigma^{v}={\left\{\Sigma_{k}^{v}\right\}}_{k=1}^{K}$.

Because this likelihood is intractable to compute and the variables $\vec{\phi_{v}}$, *z* are latent, we use the expectation maximization (EM) algorithm to optimize model parameters. EM algorithm iteratively optimizes the likelihood Eq (4). Each EM iteration consists of two steps, i.e., expectation step (E-step) and maximization step (M-step). The details are given as follows:

In E-step, we fix $\vec{\phi_{v}}$, *μ* ^*v*^ and Σ^*v*^, and then compute the expectations for *z* as:

$$\begin{array}{c} p\;(z_{n}=k|\vec{c_{n}^{v}},\vec{\phi_{v}},\mu^{v},\Sigma^{v})=\frac{\phi_{v,k}Gauss\;(\vec{c_{n}^{v}},\mu_{k}^{v},\Sigma_{k}^{v})}{\sum_{i=1}^{K}\phi_{v,i}Gauss\;(\vec{c_{n}^{v}},\mu_{i}^{v},\Sigma_{i}^{v})} \end{array}$$ (5)

In M-step, we fix the expectations of *z* obtained in E-step, and then optimize $\vec{\phi_{v}}$, *μ* ^*v*^ and Σ^*v*^ by maximizing the likelihood Eq (4). The update rules are as follows:

$$\begin{array}{c} \mu_{k}^{v}\leftarrow \frac{\sum_{n=1}^{n_{v}}p\;(z_{n}=k|\vec{c_{n}^{v}},\vec{\phi_{v}},\mu^{v},\Sigma^{v})\;\vec{c_{n}^{v}}}{\sum_{n=1}^{n_{v}}p\;(z_{n}=k|\vec{c_{n}^{v}},\vec{\phi_{v}},\mu^{v},\Sigma^{v})} \end{array}$$ (6)

$$\begin{array}{c} \Sigma_{k}^{v}\leftarrow \frac{\sum_{n=1}^{n_{v}}p\;(z_{n}=k|\vec{c_{n}^{v}},\vec{\phi_{v}},\mu^{v},\Sigma^{v})\;\vec{c_{n}^{v}}\;{\left(\vec{c_{n}^{v}}\right)}^{T}}{\sum_{n=1}^{n_{v}}p\;(z_{n}=k|\vec{c_{n}^{v}},\vec{\phi_{v}},\mu^{v},\Sigma^{v})} \end{array}$$ (7)

ϕv,k←(∑n=1nvϕv,kGauss(cnv→,μkv,Σkv)∑i=1Kϕv,iGauss(cnv→,μiv,Σiv)+β)/(∑n=1nvϕv,kGauss(cnv→,μkv,Σkv)∑i=1Kϕv,iGauss(cnv→,μiv,Σiv)+β)(nv+Kβ)(nv+Kβ) (8)

**Fig 6:**
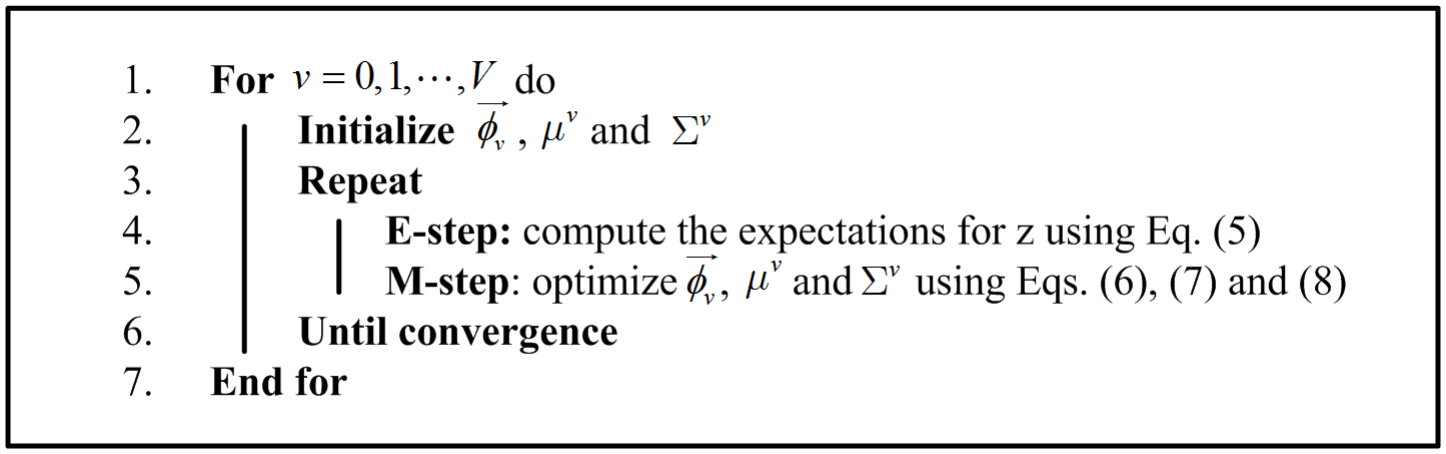
Estimation process for e-GMM.

Iterating E-step and M-step until convergence, we can obtain the optimal $\vec{\phi_{v}}$, *μ* ^*v*^ and Σ^*v*^. For clarity, we summarize the estimation process in Fig 6.

### Related work

There are some related attempts aimed at improving the spatial pooling of SPM. [27] proposes a feature and spatial covariant kernel under the histogram representation, which considers the spatial constraints against heavy cluster and occlusion. The authors of [28] combine convolutional neuron networks with the spatial pooling method. The receptive filed learning (RFL) and generalized regular spatial pooling (GRSP) proposed in [29] and [30], respectively, explore optimal pooling grids based on SPM. RFL learns adaptive grids by optimizing the pooling parameters; and GRSP allows the pooling grids in the same resolution (i.e., level) have denser or sparser distributions. Our SDP also focuses on learning more optimal pooling grids than SPM. In comparison with the above two relevant works, the advantage of SDP is to consider each visual word individually. This is more reasonable following the intuition that different visual words should obey different spatial layout distributions.

Generally, there are some other works aimed at improving spatial pooling from the part model perspective. The reconfigurable bag of words (RBoW) model [31] divides the image into a set of pre-defined sub-models, which are related to the spatial information. The visual words in RBoW have different weights for different sub-models. In a sense, this RBoW model is similar to topic models, which assign each gird of the image a “topic”. Deformable part-based models (DPM) such as [32, 33] use deformation parameters to penalize the deviation of the parts from the default locations, which are relative to the root. In comparison with these algorithms, roughly the main difference to our SDP is that they consider the spatial pooling at the grid level but SDP considers the spatial pooling at the visual word level. Particularly, in DPM deformation parameters and the part appearance models are trained jointly (i.e., latent SVMs) but in SDP the latent grids of visual words and classifiers are trained separately.

## Experiment

In this section, we evaluate the proposed SDP algorithm on three widely used datasets: Caltech-101 [17], Caltech-256 [18] and MIT-indoor-71 [37]. We use the dense SIFT descriptor to extract local features. The SIFT descriptors extracted from 16×16 pixel patches are densely sampled from each image on a grid with stepsize 8 pixels [13]. The locality-constrained linear coding (LLC) [5] algorithm is used to train the visual vocabulary, and the number of neighbors is set to 5 with the shift-invariant constraint. In this setting, five visual words are actually assigned to descriptors. For each visual word per descriptor, SDP estimates its latent grid and accumulates its word weight from LLC to this grid. To process images of different sizes, SDP normalizes the coordinates by the width and height of images. For the final image-level representation, we use the “max-pooling” combined with “*L*2 normalization” as in [5]. For SDP, the number of latent grids is set to 21, and the parameter *M* is set to 3, and the Dirichlet prior *β* is set to 1; for SPM, 1×1, 2×2 and 4×4 regular grids are used. The popular LibSVM [26] tool is used to train the classifier.

### Caltech-101

The dataset Caltech-101 collects 9144 images divided into 101 classes. The majority of images are medium resolution around 300×300 pixels and the number of images varies from 31 to 800 per class. Following the previous studies [5, 14], we train on 5, 10, 15, 20, 25 and 30 images per class and no more than 50 test images per class. For balance, all images are resized to be no larger than 300×300 pixels.

We train a visual vocabulary with 2048 visual words. Some reported results in [4, 13, 19–22, 29, 30, 34] are used as performance baselines. The experimental results are shown in Table 1. It can be seen that SDP outperforms other spatial pooling methods, e.g., about 2.5% improvements to SPM in all settings and about 2% improvements to RFL. However, a gap still exists between our SDP and the state-of-the-art algorithms, which uses more complex coding methods. This indicates that the coding method is more important for classification of dataset Caltech-101.

**Table 1:** Image classification accuracy on dataset Caltech-101. Top: comparison between algorithms using similar systems. Bottom: comparison with the state-of-the-art in the publications.

training images	5	10	15	20	25	30
LLC+SPM (*V* = 2048)	51.2	60.2	67.8	69.4	71.9	74.2
LLC+RFL (*V* = 1024) [29]						75.3
LLC+GRSP(*V* = 2048) [30]						76.7
LLC+SDP (*V* = 2048)	**53.6**	**62.8**	**69.7**	**72.8**	**75.6**	**77.1**

Lazebnik [4]			56.4			64.6
Zhang [19]	46.6	55.8	59.1	62.0		66.2
Boiman [20]	44.2	54.5	59.0	63.3	65.8	67.6
Yang [13]			67.0			73.2
Bo [21]						76.8
Bo [22]						82.5
Xie [34]	61.9	71.8	76.0	78.5		82.5

### Caltech-256

The dataset Caltech-256 collects 30,607 images divided into 256 classes, where each class contains at least 80 images. We train on 15, 30, 45 and 60 images per class and at most 50 images for testing. Similar to dataset Caltech-101, we also resize the images to be no larger than 300×300 pixels.

We train a visual vocabulary with 2048 visual words. We use some reported results in [18, 22–25, 35] as performance baselines. The experimental results are shown in Table 2. First, we observe that SDP outperforms SPM in all settings, i.e., about 3% ∼ 5% improvements. Second, SDP is competitive with the reported results, e.g., about 2% improvements against [25], and is a little lower than state-of-the-art algorithms based on more complex coding and heterogeneous features. We argue that SDP is a better and effiective spatial pooling method.

**Table 2:** Image classification accuracy on dataset Caltech-256. Top: comparison between algorithms using similar systems. Bottom: comparison with the state-of-the-art in the publications.

training images	15	30	45	60
LLC+SPM (*V* = 2048)	34.1	42.4	47.4	49.2
LLC+SDP (*V* = 2048)	**36.9**	**44.3**	**49.5**	**53.1**

Griffin [18]	28.3	34.1		
Kulkarni [25]	39,4	45.8	49.3	51.4
Arandjelovic [24]	41.2	49.5	53.9	56.8
Sanchez [23]	39.8	48.0	52.4	55.4
Kobayashi [35]	40.1		46.8	
Bo [22]	42.7	50.7	54.8	58.0

### MIT Indoor-67

The dataset MIT Indoor-67 collects 15,620 images divided into 67 indoor scene classes. We train algorithms on 80 images per class and test on 20 images per class. A visual vocabulary with 2048 visual words is trained and some reported results in [22, 30, 34, 36] are used as performance baselines.

Table 3 shows the experimental results. Also, we observe that SDP outperforms other spatial pooling methods, e.g., about 5% improvements to SPM and about 2% improvements to GRSP, and is competitive with some reported results. Altough a gap still exists between SDP and the state-of-the-art results using more complex coding methods, SPM is successful in spatial pooling.

**Table 3:** Image classification accuracy on dataset MIT Indoor-67. Top: comparison between algorithms using similar systems. Bottom: comparison with the state-of-the-art in the publications.

Algorithm	Accuracy
LLC+SPM (*V* = 2048)	43.8
LLC+GRSP (*V*) = 2048) [30]	45.2
LLC+SDP (*V* = 2048)	**48.2**

Perronnin [36]	56.2
Bo [22]	51.2
Xie [34]	46.4

### Experiments with Parameters

We investigate the influence of two significant parameters *M* and *K* on datasets Caltech-101 and Caltech-256. For dataset Caltech-101/Caltech-256, 30/60 images per class are used for training.

**Fig 7:**
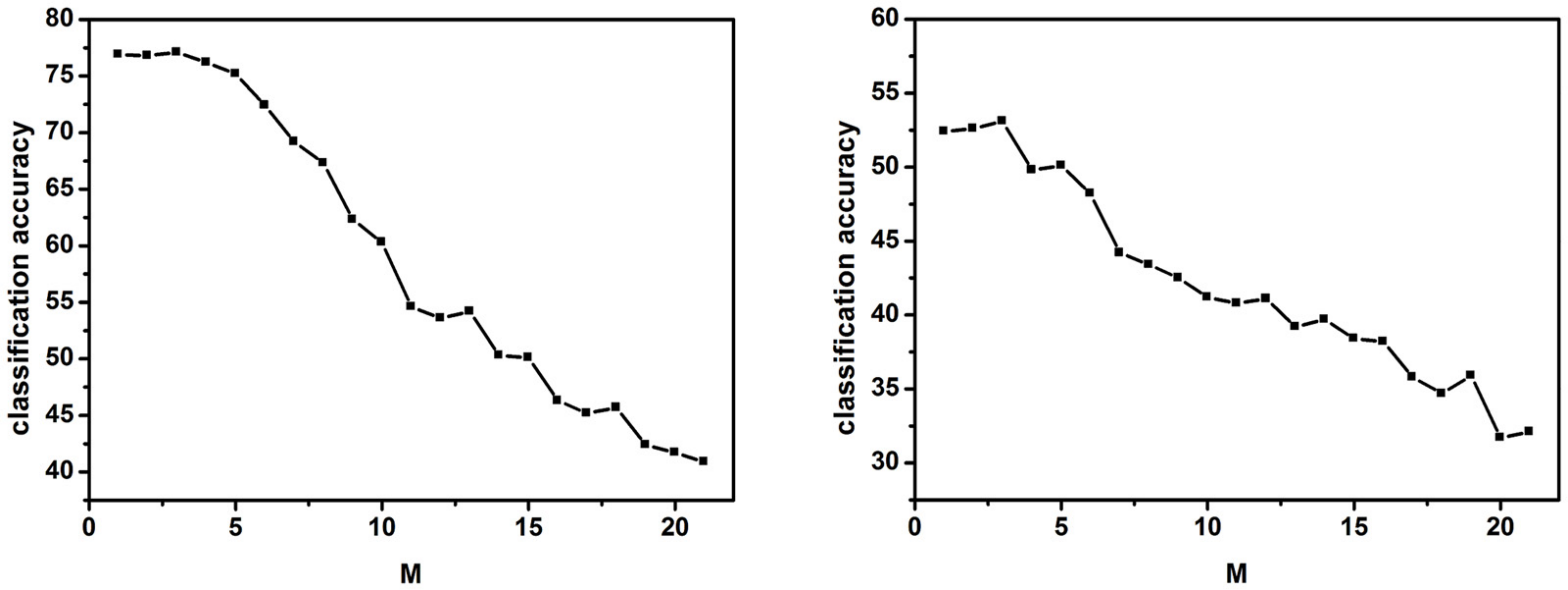
Evaluation of *M* on datasets Caltech-101 (left) and Caltech-256 (right).

We fix *K* = 21, and evaluate the classification accuracy with different *M* values over the set {1, 2, ⋯, 21}. The experimental results are shown in Fig 7. For both datasets, we observe that the results show similar trends, i.e., smaller *M* values commonly perform better than larger *M* values. For example, *M* = 3 is about 35% better than *M* = 21 on dataset Caltech-101, and *M* = 3 is about 25% better than *M* = 21 on dataset Caltech-256. That is because larger *M* values lead to dense image-level feature vectors. This reduces the discrimination of feature vectors and provides negative influence for classifiers, especially for SVMs. Besides, we observe that the performance gaps between *M* = 1, 2, 3, 4 are very small, and when *M* goes larger than 4, the performance starts to drop. In practice, value 3 performs best and is used as the default setting for the parameter *M*.

**Fig 8:**
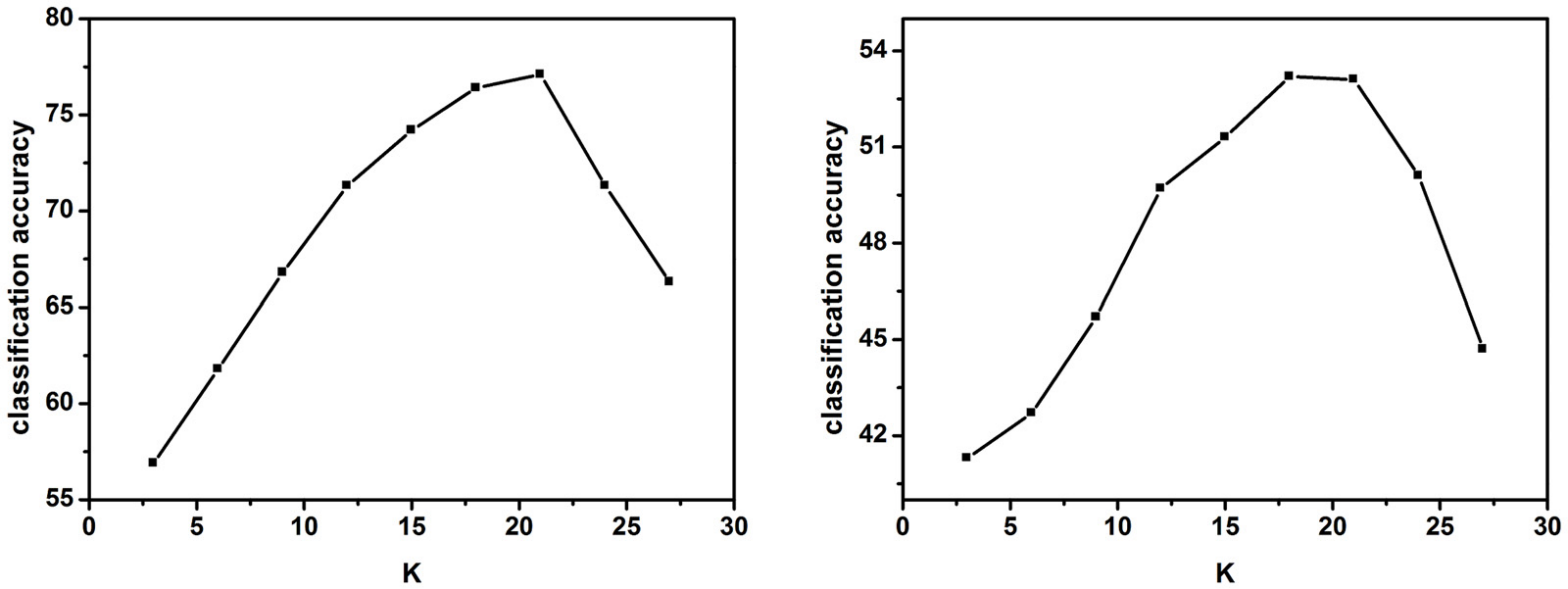
Evaluation of *K* on datasets Caltech-101 (left) and Caltech-256 (right).

We fix *M* = 3, and evaluate the classification accuracy with different *K* values over the set {3, 6, 9, 12, 15, 18, 21, 24, 27}. The experimental results are shown in Fig 8. It can be seen that relatively larger *K* values perform better than smaller *K* values, and when *K* goes larger than 21, the performance starts to drop. The best performance is achieved by *K* = 18, 21. It is interesting that it is close to the commonly used SPM setting of 1×1, 2×2 and 4×4 grids, where the total number of regular grids is 21. In practice, value 21 is used as the default setting for the parameter *K*.

## Conclusion

In this paper, we develop a novel SDP algorithm to improve the spatial pooling in the BoW model for image classification. Different from SPM, SDP algorithm individually consider each visual word. SDP is based on the proposed e-GMM model, which describes the generative process for spatial coordinates of visual word tokens. This e-GMM model assumes that for each visual word, there are some latent grids and neighborhood tokens are inclined to assign to the same grid. SDP uses e-GMM to organize flexible latent grids, and then concatenates all visual word histograms together to construct image-level feature vectors. This is more reasonable than SPM, which divides images into regular grids. We evaluate the proposed SDP algorithm on three widely used image datasets Caltech-101, Caltech-256 and MIT-indoor-67. The experimental results indicate that SDP significantly improves the performance of SPM. In our experiments we use the setting of LLC+SDP, however, this setting performs worse than some state-of-the-art algorithms using more complex coding methods. In the future, we plan to refine and apply SDP to the state-of-the-art features such as Fisher vectors [36].
